# Whole-genome sequencing data of Kazakh individuals

**DOI:** 10.1186/s13104-021-05464-4

**Published:** 2021-02-04

**Authors:** Ulykbek Kairov, Askhat Molkenov, Saule Rakhimova, Ulan Kozhamkulov, Aigul Sharip, Daniyar Karabayev, Asset Daniyarov, Joseph H.Lee, Joseph D.Terwilliger, Ainur Akilzhanova, Zhaxybay Zhumadilov

**Affiliations:** 1 Laboratory of Bioinformatics and Systems Biology, Center for Life Sciences, National Laboratory Astana, Nazarbayev University, Nur-Sultan, Kazakhstan; 2 Laboratory of Genomic and Personalized Medicine, Center for Life Sciences, National Laboratory Astana, Nazarbayev University, Nur-Sultan, Kazakhstan; 3 Columbia University, New York, USA; 4 School of Medicine, Nazarbayev University, Nur-Sultan, Kazakhstan

**Keywords:** Whole genome, Next-generation sequencing, Kazakh ethnicity, Bioinformatics analysis, Population genomics, Genome annotation, Biomedical research, Kazakhstan

## Abstract

**Objectives:**

Kazakhstan is a Central Asian crossroad of European and Asian populations situated along the way of the Great Silk Way. The territory of Kazakhstan has historically been inhabited by nomadic tribes and today is the multi-ethnic country with the dominant Kazakh ethnic group. We sequenced and analyzed the whole-genomes of five ethnic healthy Kazakh individuals with high coverage using next-generation sequencing platform. This whole-genome sequence data of healthy Kazakh individuals can be a valuable reference for biomedical studies investigating disease associations and population-wide genomic studies of ethnically diverse Central Asian region.

**Data description:**

Blood samples have been collected from five ethnic healthy Kazakh individuals living in Kazakhstan. The genomic DNA was extracted from blood and sequenced. Sequencing was performed on Illumina HiSeq2000 next-generation sequencing platform. We sequenced and analyzed the whole-genomes of ethnic Kazakh individuals with the coverage ranging from 26 to 32X. Ranging from 98.85 to 99.58% base pairs were totally mapped and aligned on the human reference genome GRCh37 hg19. Het/Hom and Ts/Tv ratios for each whole genome ranged from 1.35 to 1.49 and from 2.07 to 2.08, respectively. Sequencing data are available in the National Center for Biotechnology Information SRA database under the accession number PRJNA374772.

## Objective

Recent improvements in sequencing technology (next-generation and third-generation sequencing platforms) have sharply reduced the cost of sequencing. Next-generation sequencing has been developed in order to improve throughput and facilitate the establishment of large-scale data sets in a relatively short time [1, 2]. The 1000 Genomes Project is an international research effort to sequence a large number of people and establish the most detailed catalogue of human genetic variation [3]. There are currently different sources of complete human genomes publicly available [4, 5]. Moreover, in most of the countries the national initiatives devoted to whole-genome sequencing, such as 100 K genomes (England) have been initiated and ongoing.

Kazakhstan is the multi-ethnic and ninth largest country in the world located in Central Asia with the dominant Kazakh ethnic group [6]. Kazakhs have been strongly influenced by the nomadic lifestyle, and a long history of migration has led to admixture of Western and Asian populations, which has formed the genetic background. Thus, it is crucial to understand the genetic architecture of ethnic Kazakhs to properly investigate the genetic basis of common traits in Kazakh population. Despite the research success on comparative population studies and whole-genome sequencing of different ethnic groups, Kazakh population genomes remain absent and underrepresented in the majority of available human genome databases.

Here, we report the first complete genome sequences and analysis based on quality metrics of Kazakh ethnic individual’s generated using next-generation sequencing platform and available for further investigations. Whole-genome sequencing data obtained at high coverage (26X-32X) from four males and one female from Kazakhstan. The resulting whole genome sequences and analysis of Kazakh individuals represent an important and valuable contribution to our knowledge of the genetic landscape of the Central Asian region. Moreover these data may serve as useful resource for application in biomedicine and clinical practice to compare disease specific genetic variants with healthy/normal variants. But considering significant advancement of next-generation technologies and existence of different protocols, it is important to follow the standardized quality management rules for making efficient downstream analysis and utilization of same dataset for different purposes [7].

## Data description

Blood samples from four males and one female have been collected from Kazakh individuals living in Kazakhstan. Genomic DNA was isolated from peripheral blood using Qiagen QIAmp DNA blood mini kit. Concentration of DNA was measured using NanoDrop 2000 spectrophotometer (Thermo Fisher Scientific, USA) and Qubit Fluorimeter 2.0 (Thermo Fisher Scientific, USA). DNA quality was checked using Bioanalyzer 2100 (Agilent Technologies, USA). Paired-end DNA libraries were prepared from 1 μg of gDNA using Illumina TruSeq DNA Sample Preparation kit according to manufacturer’s protocol (Illumina, USA). Input gDNA amounts have been sheared by Covaris. End repair and 3′ adenylation steps have been performed by End repair mix and A-tailing mix, correspondingly. Indexed paired-end adapters were purified on a gel with following steps of PCR amplification and library validation for fragment size using Agilent HS DNA kit and Qubit HS DNA Assay kit. 300–400 base pairs library insert size has been selected. gDNA fragments were hybridized to the flow cell surface by TruSeq PE Cluster kit v.3 cBot HS and amplified to form clusters using Illumina cBot. High-throughput sequencing was performed by TruSeq SBS kit v.3 HS. Samples sequenced using Illumina HiSeq2000 platform to target 30-fold coverage using paired-end sequencing.

Conversion of generated bcl files to fastq format has been performed using Bcl2fastq tool. We generated 473.4 Gb of data and 4,743,332,930 short reads with average coverage 29X for five sequenced samples. The quality assessment of raw sequence reads was performed with FastQC v.0.11.7 [8]. Reads were aligned and assembled on the human reference genome (NCBI GRCh37, hg19) and reference mitochondrial DNA rCRS (NC_012920) using Burrows-Wheeler Aligner v.0.7.12 [9]. Alignments corresponding to specific samples were merged into a single BAM file (Data files 1 to 5—Table 1) and marked for duplicates using Picard tools v.1.130. The alignment quality was assessed using SAMtools v.1.2 [10].

**Table 1:** Overview of data files/data sets

Label	Name of data file/data set	File types (file extension)	Data repository and identifier (DOI or accession number)
Data file 1	SRX2563808/PRJNA374772	BAM file (.gz)	NCBI SRA https://identifiers.org/ncbi/insdc.sra:SRX2563808 [15]
Data file 2	SRX2563806/PRJNA374772	BAM file (.gz)	NCBI SRA https://identifiers.org/ncbi/insdc.sra:SRX2563806 [16]
Data file 3	SRX2563805/PRJNA374772	BAM file (.gz)	NCBI SRA https://identifiers.org/ncbi/insdc.sra:SRX2563805 [17]
Data file 4	SRX2563804/PRJNA374772	BAM file (.gz)	NCBI SRA https://identifiers.org/ncbi/insdc.sra:SRX2563804 [18]
Data file 5	SRX2563803/PRJNA374772	BAM file (.gz)	NCBI SRA https://identifiers.org/ncbi/insdc.sra:SRX2563803 [19]
Data file 6	SuppTable_S1-SeqAndMappingSummary.docx	Word file (.docx)	https://github.com/LabBandSB/wgs_pipeline_on_hg19/raw/master/SuppTable_S1-SeqAndMappingSummary.docx [20]
Data file 7	SuppTable_S2-MappingOf UnmappedReadsToNCBIscaffolds.docx	Word file (.docx)	https://github.com/LabBandSB/wgs_pipeline_on_hg19/raw/master/SuppTable_S2-MappingOf%20UnmappedReadsToNCBIscaffolds.docx [21]

From 98.85 to 99.58% base pairs were totally mapped with properly mapped 99.06% on average. Het/Hom and Ts/Tv ratios for each whole genome ranged from 1.35 to 1.49 and from 2.07 to 2.08, respectively (Data file 6—Table 1). As a measure of the quality of our whole-genome sequencing data, human genome studies particularly from the 1000 Genomes project have been showing that for whole genomes, a Ts/Tv ratio of around 2–2.1 is generally a good quality ratio [3, 11, 12]. Het/Hom ratio also can be used for whole genome sequencing quality metric, but highly depends on ancestry and varies in different populations [13, 14]. Additionally we mapped unmapped sequencing reads to unanchored NCBI human scaffolds and reported that from 411 to 629 unmapped sequencing reads were mapped what corresponds from 0.021 to 0.037 percentage from total number of unmapped sequences (Data file 7—Table 1). The rest of unmapped sequencing reads may suggest for ethnical or individual uniqueness and could be other factors affecting the mapping such as contamination or sequencing errors.

Here we described high-coverage whole genome sequencing data and analysis based on quality metrics of Kazakh individuals representing a valuable resource for the research community complementing the world’s genomics map on a global population scale.

## Limitations

Small sample size of described individuals as well as the only next-generation sequencing approach applied without replication performed using the other technologies such genotyping by microarray or third-generation sequencing are limitations of our work.

## Data Availability

The data described in this Data note can be freely and openly accessed on NCBI SRA Database under PRJNA374772 accession number. Please see Table 1 and references [15–21] for details and links to the data as well as sequencing and mapping summary and mapping of unmapped reads to NCBI unanchored scaffolds summary.

## Abbreviations

DNA: Deoxyribonucleic acid
gDNA: Genomic DNA
Het/Hom: Heterozygous/Homozygous
Ts/Tv: Transitions/Transversions
PCR: Polymerase chain reaction
NCBI: National Center for Biotechnology Information
SRA: Sequence Read Archive
BAM: Binary alignment map
